# Therapeutic strategies for oral lichen planus: State of the art and new insights

**DOI:** 10.3389/fmed.2022.997190

**Published:** 2022-10-04

**Authors:** Dario Didona, Raffaele Dante Caposiena Caro, Antonio Manuel Sequeira Santos, Farzan Solimani, Michael Hertl

**Affiliations:** ^1^Department of Dermatology and Allergology, Philipps-Universität Marburg, Marburg, Germany; ^2^Dermatology Clinic, Hospital Maggiore of Trieste, University of Trieste, Trieste, Italy; ^3^Department of Dermatology, Venereology and Allergology, Charité-Universitätsmedizin Berlin, Berlin, Germany; ^4^Berlin Institute of Health at Charité - Universitátsmedizin Berlin, BIH Biomedical Innovation Academy, BIH Charité Clinician Scientist Program, Berlin, Germany

**Keywords:** erosive lichen planus, oral mucosa, oral lichen pianus, treatment, biologic therapies

## Abstract

Oral lichen planus (OLP) is a chronic inflammatory disease of the oral mucosa. Several clinical subtypes of OLP have been reported, including the reticular and erosive one. On the one hand, reticular OLP is usually asymptomatic and is characterized by white streaks surrounded by well-defined erythematous borders. On the other hand, erosive OLP shows ulcerations and erosions surrounded by erythematous mucosa. While reticular OLP is relatively easy to control, erosive OLP is extremely painful and refractory to therapies, limiting the quality of life of the patients. In addition, treating erosive OLP is extremely tricky, and a gold standard treatment has not yet been established. However, several therapeutic approaches have been reported as effective, including systemic corticosteroids, systemic retinoids, and anti-interleukin (IL)-17/anti-IL-23 drugs. Indeed, our group and other several authors reported the effectiveness of anti-IL17, anti-IL12/23, and anti-IL23 agents in refractory OLP, highlighting the urgency of clinical studies on the use of anti-IL agents in OLP patients. In this paper, we reviewed the English- and German-language literature about therapeutic strategies for treating OLP, focusing on new systemic therapies for erosive OLP.

## Introduction

Lichen planus (LP) is a chronic inflammatory disease that can affect skin, mucous membranes, and skin appendages. The prevalence of LP in the general population is up to 1.27% (1). LP can occur at any age, without sex or racial preferences (1, 2). Mucosal LP (MLP) shows a prevalence of 0.89% and it is more commonly diagnosed in the female population (1, 2). Oral LP (OLP) represents the most common form of MLP and can be diagnosed as isolated disease or in association with cutaneous, scalp, nail, or mucosal involvements, including the genital, gastrointestinal, and ocular mucosa. Several therapies can be used to treat the different clinical variants of LP, although some subtypes of OLP are characterized by a refractory clinical course. Therefore, new therapeutic strategies, including the use of interleukin (IL) inhibitors and Janus kinase inhibitors (JAKI), have been proposed as possible therapies in difficult cases.

## Clinical presentation and follow-up

**Figure 1:**
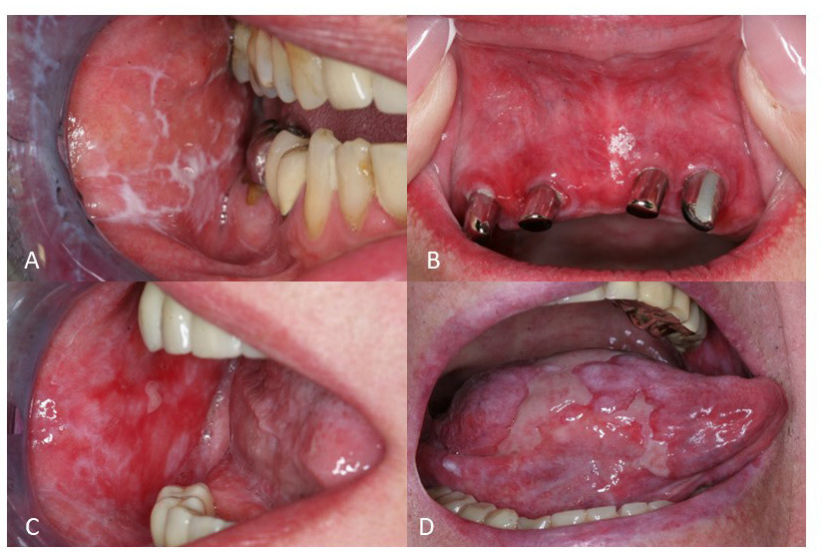
Clinical manifestation of oral lichen planus (OLP). **(A)** Reticular OLP with characteristic Wickham's striae **(B)** Erythema in a female patient with OLP **(C)** Multiple erosions on the left buccal mucosa in a patient with erosive OLP **(D)** Extreme painful ulcerations of the tongue in a patient with ulcerative OLP.

Several clinical subtypes of OLP have been described, including reticular, plaque-like, papular, erosive, ulcerative, atrophic, and bullous OLP (Figures 1A–D) (3, 4). Oral involvement has been reported in up to 90% of the patients with cutaneous LP (5). Approximately 15% of OLP patients develop cutaneous lesions and up to 20% of OLP patients show genital lesions (5). Several triggers, such as traumas, dental procedures, and cigarette smoking can exacerbate OLP (5). Reticular OLP is the most common subtype, and it is usually asymptomatic. It is characterized by white streaks surrounded by well-defined erythematous borders. Reticular OLP can eventually evolve into the other subtypes, including the erosive one. Plaque-like OLP is characterized by homogenous white patches. In this case, a malignant leukoplakia must always be ruled out. Furthermore, it has been observed, that this variant is more prevalent in tobacco smokers (6). Clinical features of erosive OLP are represented by atrophic or erythematous ulcerations and erosions. Typically, it shows a multifocal pattern of distribution. The atrophic subtype has similarities to the erosive subtype, but shows more prominent atrophic lesions on a background of erythema. Moreover, atrophic OLP primarily affects the gingiva and the buccal mucosa in the posteroinferior areas adjacent to the second and third molar teeth (3).

A regular screening for oral cancer in OLP is recommended. Indeed, several risk factors for malignant transformations in OLP have been reported, including erosive clinical phenotype, involvement of the tongue, female gender, and advanced age (7, 8). At this regard, Fitzpatrick et al. found that 85 (1.09%) of 7806 OLP patients and 4 (3.2%) of 125 patients with oral lichenoid lesions developed an oral squamous cell carcinoma (9). Furthermore, Georgakopoulou et al. reported a malignant transformation rate in OLP of 12.5% (10). In addition, a recent systematic review detected a transformation rate of 1.37 for OLP (11). Therefore, an annual monitoring to detect early malignant lesions is strongly recommended and it should be performed by oral medicine specialists (7, 8).

## Pathogenesis of oral lichen planus

Antigen-specific and non-specific mechanisms are involved in the pathogenesis of OLP (12, 13). On the one hand, antigen presentation by keratinocytes and Langerhans cells to CD4+ helper and CD8+ cytotoxic T lymphocytes leads to their activation (12, 13). The activated helper T cells produce IL-2 and interferon (IFN)-gamma and lead to the proliferation and activation of cytotoxic T lymphocytes, which cause the apoptosis of basal keratinocytes and the degeneration of basal epithelial cells typically found in OLP lesions (12–14). Furthermore, Solimani et al. strongly suggested that IL-17 plays a critical role in the pathogenesis of OLP (15). Indeed, IL-17 induces chemokine production from different cells, including endothelial cells, macrophages, and keratinocytes, leading to tissue remodeling and recruitment of pro-inflammatory cells (16). Moreover, IL-17 release activates a pro-inflammatory cascade that leads to recruitment of T lymphocytes (15). On the other hand, mast cell degranulation and production of tumor necrosis factor (TNF)-alpha and chymase play a role in the pathogenesis of OLP. Indeed, TNF-alpha is involved in the migration of T cells to migrate from the capillaries into the surrounding extracellular matrix. In addition, chymases activate the matrix metalloproteinase-9, which subsequently destroys the basal membrane and leads to the migration of CD8+ cytotoxic T lymphocytes into the mucosal lesions (12, 13). Therefore, OLP is considered as a T-lymphocyte-mediated chronic inflammatory mucosal disease. However, some authors suggested that autoimmunity can play a role in OLP pathogenesis, pointing out that CD8+ cytotoxic T lymphocytes can recognize antigens associated with major histocompatibility complex (MHC) class I on lesional keratinocytes (17).

## Diagnosis

The diagnosis of OLP relies on clinical and histological features. Clinical features of OLP are usually sufficient to establish the diagnosis, especially if patients show also typical skin lesions, such as Wickham's striae and symmetric, purplish, flat, polygonal, itchy papules on the extremities (18, 19). However, a biopsy of oral lesions is recommended to confirm the clinical diagnosis and exclude malignancy (18, 19). Histologically, OLP typically shows ortho- or parakeratosis, absence of epithelial dysplasia, apoptotic keratinocytes (Civatte's bodies) in the basal layer, a well-defined, band-like lymphocytic infiltration limited to the superficial part of the connective tissue, and vacuolization of the basal cell layer (4, 18). Direct immunofluorescence can be useful to exclude bullous diseases of the oral mucosa, such as pemphigus vulgaris, paraneoplastic pemphigus, and bullous pemphigoid (20, 21). Patch tests should be performed to rule out type IV allergic reactions in patients with a medical history that suggests allergies to dental materials.

## Therapies

Several therapeutic options can be used in OLP. Non-erosive OLP can be usually treated with topical potent corticosteroids (CS) (e.g., clobetasol propionate 0.05%) (19). Intralesional injection of triamcinolone can be useful in erosive OLP (19). In case of severe erosive OLP or refractory forms, several systemic therapies have been proposed as effective, including systemic CS, apremilast, hydroxychloroquine (HCQ), and systemic retinoids (5, 19). Here, we report the topical and systemic therapies of OLP, focusing on the treatment of erosive OLP. For practical purposes and easier consultation for clinicians, we reported the therapies in alphabetical order in the text, while in the Table 1 we finally listed the recommended therapies for OLP based on the review of the literature and the clinical experience of the authors (Table 1).

**Table 1:** Recommended therapies for oral lichen planus*^i^*.

**Leading clinical phenotype**	**Topical therapy**	**Systemic therapy**		
Non-erosive OLP	- Topical corticosteroids	Usually not necessary		
	- Intralesional corticosteroids			
	- Tacrolimus 0.1%^*^			
		**First line**	Second line^*^	Compassionate use^*^
Erosive OLP	- Topical corticosteroids - Tacrolimus 0.1%^*^ - PDT^*^	- Oral corticosteroids - Corticosteroids i.v. - Alitretinoin^*^	- Hydroxychloroquine - Methotrexate - Apremilast - Azathioprine	- Sekukinumab - Guselkumab - JAKI

JAKI, Janus-Kinase inhibitors; OLP, oral lichen planus; PDT, photodynamic therapy.

* Off-label in Germany.

i The therapies are listed in order of recommendation according to the experience of the authors.

## Apremilast

Apremilast is an oral phosphodiesterase type 4 inhibitor approved for the management of psoriasis and psoriasis arthritis. It reduces the production of TNF-alpha, IFN-gamma, IL-2, IL-5, IL-8, and IL-12, which contribute to the pathogenesis of OLP. The effective use of apremilast in erosive OLP was reported firstly only in case reports and case series (22, 23). Furthermore, a multicentric, retrospective study on 11 OLP patients (8 of them with a coexistent cutaneous LP) was recently published (24). In this study, the authors reported that 55% of patients had an improvement of their symptoms at week 12 (24). Reasons for therapy discontinuation were progression of disease in five patients (45%), adverse events in three patients (27%), and remission of disease in one (9%) patient (24). The authors concluded that apremilast was effective in some of patients, making its use in recalcitrant cases a possible therapeutic option (24).

## Azathioprine

Azathioprine (AZA) has been used in several skin diseases, such as pemphigus vulgaris, bullous pemphigoid, and pyoderma gangrenosum. AZA was successfully used as steroid sparing therapy only in a few patients with erosive OLP (25, 26). Indeed, Verma et al. reported a good improvement in four patients with exclusive erosive OLP and in two patients with diffuse skin LP and OLP on AZA 50 mg twice daily orally (about 2 mg/kg day), for a period varying from three to seven months (26). Therefore, the use of AZA in OLP may be recommended as off-label therapy in OLP.

## Biologics

Several biologic therapies have been used in patients with refractory OLP, including anti-CD2, anti-TNF-alpha, anti-IL2, anti-IL17, anti-IL12/23, and anti-IL23 drugs (Table 2) (15, 27). Several cases of OLP treated with anti-TNF-alpha agents have been reported. An improvement of erosive OLP was reported in a patient treated with etanercept (28). Furthermore, a patient with a severe orogenital LP was successfully treated with infliximab (27) and two other patients with a severe orogenital involvement were treated with adalimumab (29, 30). However, emerging data suggest that TNF-alpha inhibitors may trigger OLP (31, 32). Alefacept, a T-cell modulator approved by the US Food and Drug Administration for the treatment of adult patients with plaque psoriasis, has been reported as effective therapy in patients with OLP (33, 34). In particular, Chang et al. described two patients with concomitant OLP and genital lesions in a small case series, who responded to treatment with alefacept (33). Anti-IL17, anti-IL12/23, and anti-IL23 agents have been successfully used in several patients with OLP (15, 27). In a previous report, our group described a massive improvement in patients with OLP after biologic therapy. In particular, one patient was treated with ustekinumab, one with guselkumab, and three with secukinumab (15). The clinical improvement was linked to a strong reduction of the Th1 and Th17/Tc17 cellular mucosal infiltrate, suggesting that IL-17-producing T cells play a pivotal role in OLP (15). Furthermore, Ismail et al. reported the successfully use of tildrakizumab in refractory OLP (6). In addition, some cases of OLP were successfully treated with rituximab (35–37). However, in a case series was reported a failure or a transient minimal improvement of OLP after rituximab (38).

**Table 2:** Patients with oral lichen planus treated with biologics.

**Drug**	**Number of patients**	**Treatment period**	**Observation period**	**Comment**
Adalimumab (29)	1	50 weeks	50 weeks	Clinical improvement
Adalimumab (30)	1	12 weeks	12 weeks	Complete healing
Alefacept (34)	2	12 weeks	32 weeks	Clinical improvement
Alefacept (33)	2	12 weeks	12 weeks	Clinical improvement
Etanercept (28)	1	10 weeks	17 weeks	Clinical improvement and pain relief after etanercept; disease recurrence after agent discontinuation
Guselkumab (15)	1	30 weeks	30 weeks	Complete healing
Infliximab (27)	1	6 months	6 months	Clinical improvement
Rituximab (37)	1	4 weeks	10 months	Clinical improvement; relapse after 10 months
Rituximab (35)	2	14 months	14 months	Remission lasted until 8 months
Rituximab (38)	5	4 months	9 months	Clinical improvement
Secukinumab (15)	3	12–48 weeks	12–48 weeks	Complete healing
Tildrakizumab (16)	1	28 weeks	28 weeks	Complete healing
Ustekinumab (15)	1	48 weeks	48 weeks	Complete healing

## Calcineurin inhibitors

The use of topical calcineurin inhibitors, such as tacrolimus and pimecrolimus, in OLP is extremely diffuse in the clinical practice, although more placebo-controlled, randomized studies are needed to evaluate effectiveness and safety of topical calcineurin inhibitors in comparison to topical CS. In a recent meta-analysis, Sun et al. concluded that topical tacrolimus 0.1% should be the first choice within the group of topical calcineurin inhibitors for the short-term treatment of recalcitrant OLP (39). Although tacrolimus showed a higher incidence of local adverse events, such as transient burning sensation, in comparison to topical CS in the short term treatment, the local adverse reactions were significantly reduced after the resolution of the initial erosion (39). Regarding the use of tacrolimus 0.03%, only a randomised clinical trial (RCT) was reported in the literature (39). Therefore, more trials are needed to determine whether tacrolimus 0.03% is as effective as topical CS. Furthermore, Sun et al. concluded that, because of the limited RCT on pimecrolimus, tacrolimus 0.1% should be preferred to pimecorlimus in recalcitrant OLP (39). However, Volz et al. reported a significant reduction in oral erosions with topical pimecrolimus 1% compared to placebo in a prospective randomized double-blind vehicle-controlled study (40). In addition, Gorouhi et al. in a comparative study on 40 OLP patients focusing on efficacy and safety of pimecrolimus 1% cream vs. triamcinolone acetonide 0.1% paste concluded that both treatments improved the symptoms, although pimecrolimus induced burning sensation in two patients, while in the triamcinolone group no side-effects were reported (41).

Because of the possible carcinogenic effect of topical calcineurin inhibitors (42, 43) a regular screening for oral cancer should be recommended. In addition, the continued application of topical calcineurin inhibitors should be avoided if the inflammatory activity persists (5).

## Corticosteroids

Topical CS represent the first-line approach in OLP. In particular, clobetasol propionate 0.05% is often used as first therapy (5). In addition, triamcinolone, betamethasone, fluocinonide, fluticasone, dexamethasone, and prednisolone in different topical forms, such as ointment, oral suspension, aqueous solution, mouthwash, and adhesive paste, have been proven to be effective and safe (5). In a recent phase II RCT, a novel mucoadhesive clobetasol patch (Rivelin^®^ -CLO) was tested on patients with erosive OLP (44). An improvement in OLP symptoms was reported in the verum group (25/32) compared to the placebo group (11/30), (*p* = 0.012) (44). The authors concluded that Rivelin^®^ -CLO patches were superior to placebo, demonstrating statistically significant objective and subjective improvement and a favorable safety profile (44). Intralesional injection of CS, such as triamcinolone acetonide, hydrocortisone, dexamethasone, and methylprednisolone, are effective in erosive OLP, but this approach is extreme painful for the patient and only a few erosions can be treated in each session (44, 45).

Oral CS, such as dexamethasone or prednisone, are commonly prescribed in case of recalcitrant OLP. Usually, oral prednisone (0.5 mg/Kg) for 4–6 weeks is used (46). The side effects of prolonged oral CS therapy can be severe and include muscle weakness, sleep disorders, weight gain, pathologic fractures, anemia, acne, striae rubrae, and menstrual abnormalities (47). To overcome or minimize these side effects, a new concept of oral mini-pulse therapy was proposed (48). Indeed, Malhotra et al. compared a mini-pulse therapy regimen (5 mg betamethasone orally on two consecutive days per week) to triamcinolone acetonide 0.1% paste in patients with OLP (48). The authors reported that the clinical response was similar in both groups, but the patients on oral betamethasone showed an earlier clinical improvement and the side-effects (e.g. facial edema, headache, and muscular weakness) were mild, transient, and reversible (48).

## Cyclosporine

Cyclosporine (CsA) is a calcineurin inhibitor, used as an immunosuppressant medication. Systemic CsA is effective in the treatment of many inflammatory dermatoses. However, in OLP its systemic use is reported only in some case reports (49). Furthermore, because of its adverse effects, including hypertension, dysregulation of the renal function, and gingival hyperplasia, systemic CsA is not recommended as routine therapy in OLP (49, 50).

In two double-blind, placebo-controlled trials, the efficacy of topical CsA in OLP was demonstrated (51, 52). In addition, in one small study, topical CsA solution 100 mg/ml showed a better clinical improvement after eight weeks in comparison to triamcinolone solution 0.1% (53). However, in a recent study, dexamethasone solution 2 mg/5 ml was found to be significantly better than CsA solution 100 mg/ml in reducing the clinical symptoms (54). Furthermore, in a randomized, comparative, double-blind study on 40 patients, topical clobetasol was more effective in comparison to topical CsA in inducing a clinical improvement (55). In addition, the costs of a therapy with topical CsA is five times higher than one with clobetasol (55).

## Dapsone

Dapsone is used in combination with clofazimine and rifampicin for the treatment of leprosy. Furthermore, it is used in dermatology to treat lymphocyte-mediated inflammatory diseases. Regarding OLP, dapsone was reported as useful only in two case reports (56, 57). Therefore, because of its important hematological adverse effects, including methemoglobinemia and hemolytic anemia, and the several alternative therapeutic options, dapsone cannot be recommended as routine therapy in OLP.

## Hydroxychloroquine

HCQ is worldwide used as an anti-malarial agent. Because of its immunomodulatory action, HCQ is widely used in dermatology as therapy for different diseases, including systemic lupus erythematosus, polymorphous light eruption, and dermatomyositis.

In 1993, Eisen reported an overall response rate of 90% in a retrospective, non-randomized study in nine erosive OLP patients after HCQ (58). In a recent prospective clinical trial on 45 patients with erosive OLP, HCQ 200 mg p.o. twice daily as monotherapy was reported as effective and safe (59). In addition, Yeshurun et al. reported a moderate to marked improvement in 57% and a complete remission in 24% patients with erosive OLP on HCQ 400 mg/day p.o. as monotherapy (60).

HCQ is generally well tolerated with minor gastrointestinal symptoms (e.g. nausea, vomiting, and diarrhea) and neuromuscular symptoms (e.g. headaches, myalgia, and fatigue) (61). Some of the infrequent adverse effects of a long-term monotherapy (e.g. agranulocytosis, retinopathy, and cutaneous hyperpigmentation) are reversible after drug discontinuation (61).

## Janus kinases inhibitors

JAKI are emerging as a new class of drugs, which can be used in several dermatological diseases, including atopic dermatitis and alopecia areata (62, 63). In OLP the use of JAKI is limited to case reports. Three OLP patients were successfully treated with JAKI, one of them with baricitinib and two others with upadacitinib (64–66).

## Lasers

Lasers represent a non-pharmacological and non-invasive alternative option for the treatment of OLP. Low-level laser (LLL) includes various light sources such as helium neon (633 nm), ruby (694 nm), and argon (488 and 514 nm). In a RCT, a comparative evaluation of LLL and CO2 laser was performed (67). Both methods were reported as effective in the treatment of OLP, but LLL led to a more rapid improvement of lesions than CO2 lasers (67). The effectiveness of CO2 laser was also reported by Van der Hem et al. (68) and by Dalirsani et al. (69). In comparison to photodynamic therapy (PDT), LLL was less effective in a study conducted on 45 OLP patients (70). A comparison between topical CS and LLL was also performed, showing variable results (71). Indeed, in one study on 34 OLP patients, clobetasol gel 0.05% was more effective than LLL, while, in another one on 42 OLP patients, LLL showed a better effectiveness in comparison to clobetasol gel 0.05% (71, 72). Furthermore, dexamethasone solution and triamcinolone paste 0.1% showed higher efficacy than LLL (71, 73). The positive effects of LLL on erosions and ulcerations in OLP could be explained by its biological activity on different cells, such as fibroblasts and epithelial cells, which play a pivotal role in the wound healing process (72).

## Methotrexate

Methotrexate (MTX) is a folate antimetabolite that inhibits DNA synthesis, repair, and cellular replication. MTX can be administered orally or subcutaneously and is useful in several inflammatory dermatoses, including psoriasis and bullous pemphigoid. Several dose-related adverse effects have been reported in patients on MTX, including stomatitis, gastrointestinal problems, and cutaneous rash. Oral MTX was used in a prospective open trial in patients with unresponsive OLP (74). The authors reported a partial response in 83.3% of the patients (74). In a recent prospective, observational study, oral MTX in combination with triamcinolone 0.1% oral paste was reported as more effective in comparison to oral MTX and triamcinolone 0.1% oral paste as monotherapy in patients with severe OLP (75). The authors concluded that MTX can be considered as first line options in patients with moderate to severe OLP, either alone or in combination with topical triamcinolone (75).

## Mycophenolate mofetil

Mycophenolate mofetil (MMF) is a prodrug of mycophenolic acid, an inhibitor of the two isoforms of inosine monophosphate dehydrogenase. Mycophenolic acid has potent cytostatic effect mainly on lymphocytes. Therefore, MMF represents a valid therapy in several autoimmune skin diseases. Gastrointestinal side effects and reduction of peripheral leukocytes are reported as common side effects. On the one hand, the efficacy of MMF in OLP was not tested in double-blind, placebo-controlled trials (49). On the other hand, its effective use was reported in some case reports and in two retrospective case series (76–78). In conclusion, weak evidence exists so far to support the routinary use of MMF in OLP (49, 50).

## Photodynamic therapy

PDT combines the use of a photosensitive agent and a harmless light source with a particular wavelength. PDT is mainly used to treat non-melanoma skin cancers (79). Recently, the use of PDT has been growing as non-invasive therapy for OLP (80). Furthermore, PDT can be used as monotherapy or in combination with other treatment options (81). PDT with 5% methylene blue as photosensitizer was effectively used in a cohort of 20 patients with a long-standing OLP (82). Moreover, it was reported that the effectiveness of PDT depends on the localization of the lesion and is particularly reduced around the area of the masticatory oral mucosa (83). A decrease of CD4+, CD8+ and IL-17+ cells in the oral mucosa affected by OLP has been reported after PDT (80). Furthermore, a reduction of CD4+CD137+, CD8+CD137+, and IL-17+ T cells has been reported in peripheral blood after PDT in OLP patients (80). Regarding the comparison of PDT with topical CS, mixed results have been reported in clinical studies (71). On the one hand, PDT was reported as more effective over dexamethasone mouthwash (70); on the other hand, dexamethasone mouthwash showed a better effectiveness in comparison to PDT (84). Furthermore, two other studies showed a similar efficacy of PDT in comparison to dexamethasone mouthwash and triamcinolone paste 0.1% (71). In comparison to LLL, PDT was more effective in a study conducted on 45 OLP patients (70). Adverse events after PDT include erythema, pain, edema, and contact dermatitis at the site of application of the photosensitizer (71).

## Retinoids

Retinoids are derivative of vitamin A and have been widely used for treatment of acne and photoaging because of their activity in blocking inflammatory mediators and reducing keratinization of epithelial cells (85). Topical retinoids (e.g., tretinoin, tazarotene, and isotretinoin) are extremely irritating and cannot be generally recommended for OLP. However, Boisnic et al. reported a significant improvement of the clinical features in OLP patients treated with topical tretinoin 0.05% twice a day compared to placebo (86). Furthermore, Kar et al. reported that topical tretinoin 0.05% was as effective as betamethasone 0.05% in OLP patients (87). However, Buajeeb et al. described a better improvement of clinical features in patients with erosive OLP on fluocinolone 0.1% compared to the patients on tretinoin 0.05% (88). In a double-blind study on 20 patients with OLP, the use of 0.1% isotretinoin gel was compared to placebo (89). The authors reported that in the group on 0.1% isotretinoin gel four patients showed almost a complete healing of the lesions, whereas the other six showed an improvement of the lesions (89).

Systemic retinoids (e.g., acitretin, alitretinoin, and isotretinoin) are teratogenic. Therefore, in fertile female patients adequate contraception throughout the therapy and after its discontinuation is required. A prospective open-label single arm pilot study reported the efficacy and tolerance of alitretinoin (30 mg daily) in ten patients with severe OLP (90). In a retrospective study on OLP patients, Alseneid et al. evaluated the efficacy and safety of acitretin and alitretinoin (91). The authors concluded that alitretinoin should be preferred to acitretin because of its efficacy, tolerability, and better teratogenic profile (91). Regarding isotretinoin, its systemic use was reported only in a clinical study on six patients and in some case reports (92, 93). The use of oral acitretin in OLP has not been reported in the literature so far.

## Perspectives

The use of anti-IL-17, anti-IL-12/IL-23, and anti-IL-23 monoclonal antibodies was reported as extremely effective in refractory erosive OLP (2). At this regard, an open label, parallel, randomized, multi-center, phase II trial to evaluate the efficacy, safety, and tolerability of guselkumab in patients with OLP is now ongoing (EudraCT Number: 2021-000271-36). In addition, a phase II study to evaluate the efficacy, safety, and tolerability of secukinumab 300 mg over 32 weeks in clinical variants of LP, including OLP, was recently concluded (EudraCT number 2019-003588-24). At the present time, no other clinical trials have been reported in the European and US register regarding the use of systemic agents in OLP.

## Conclusion

Different clinical forms of OLP have been described in the literature. In case of mild or moderate involvement of the oral mucosa, a therapy with topical CS or topical calcineurin inhibitors usually leads to a clinic improvement. However, erosive/ulcerative OLP represents a challenge for clinicians. Indeed, erosions and ulcerations are usually refractory to topical therapies and even to systemic in-label therapies. Therefore, more RCT should be conducted to identify effective alternative therapies for OLP patients with erosive/ulcerative clinical features. In our experience, anti-IL-17, anti-IL-12/IL-23, and anti-IL-23 monoclonal antibodies represent an effective and safe alternative therapy in refractory erosive/ulcerative OLP.

## Author contributions

Conceptualization: DD and RC. Review and editing: MH. Original draft: All authors. All authors contributed to the article and approved the submitted version.

## Funding

This work has been financially supported by PEGASUS (FOR 2497). FS is participant in the BIH Charité Clinician Scientist Program funded by the Charité - Universitätsmedizin Berlin and the Berlin Institute of Health at Charité (BIH).

## Conflict of interest

Author MH has received honoraria from Novartis, Sanofi, Celgene and unrestricted grants from Biotest, Janssen Cilag, and Topas during the last 3 years. The remaining authors declare that the research was conducted in the absence of any commercial or financial relationships that could be construed as a potential conflict of interest.

## Publisher's note

All claims expressed in this article are solely those of the authors and do not necessarily represent those of their affiliated organizations, or those of the publisher, the editors and the reviewers. Any product that may be evaluated in this article, or claim that may be made by its manufacturer, is not guaranteed or endorsed by the publisher.
